# Supplementary material for: Evanescent Absorption Spectroscopy of Transition Metal Dichalcogenide Materials Using Guided Photoluminescence in Si3N4 Photonic Waveguides
Source: ACS Appl Opt Mater. 2026 Sep 10;4(9):2765–73. doi: 10.1021/acsaom.6c00415 (PMC13621805; doi:10.1021/acsaom.6c00415)
Supplement: Supplementary file 1 [file ot6c00415_si_001.pdf]

Supporting Information

Evanescent absorption spectroscopy of transition metal dichalcogenide materials using guided photoluminescence in Si_3N_4 photonic waveguides

Víctor Gonzalez-Morote[1], Marianna Sledzinska[2], Arkadiusz P. Gertych[3], Syed Hassan Abbas Jaffery[3], Mariusz Zdrojek[3], Daniel Navarro-Urrios[1*]*

[1] MIND-IN2UB, Department of Electronics and Biomedical Engineering, University of Barcelona, 08028 Barcelona, Spain

[2] Catalan Institute of Nanoscience and Nanotechnology (ICN2), CSIC and BIST, Campus UAB, Bellaterra, 08193 Barcelona, Spain

[3] Faculty of Physics, Warsaw University of Technology, Koszykowa 75, 00-662, Warsaw, Poland

Corresponding authors: vgonzalezmorote@ub.edu; dnavarro@ub.edu

S1. Optical setup and excitation geometries

The complementary top-pumping scheme implemented in the waveguide characterization setup, briefly described in Section 2 of the main text, is sketched in Fig. S1. It uses a 491 nm continuous-wave laser (Cobolt Calypso), whose beam was first expanded by a factor of 5 using a confocal two-lens telescope with focal lengths of 25 mm and 125 mm. This produced a 3 mm collimated beam, whose diameter was verified using the knife-edge technique.[1,2] A concave cylindrical mirror (focal length 100 mm) then folded the beam onto the chip and focused it along one axis to a 10 µm line while leaving the orthogonal axis unfocused, thereby forming a 10 µm × 3 mm stripe oriented along the waveguide. The folded beam reaches the sample at an incidence angle of 55.5° with respect to the surface normal. Consequently, the illuminated area is increased by a factor of 1.77 relative to the nominal stripe area, and the photon-flux density at the sample surface is reduced by a factor of cos(θ) = 0.57. The alignment of the excitation stripe with respect to the target waveguide was monitored from above using a microscope objective and a CCD camera.

Guided photoluminescence (PL) generated by excitation of the TMD material deposited on the waveguides and emerging from the output facet was collected in a manner similar to that described for Fig. 1 of the main text.

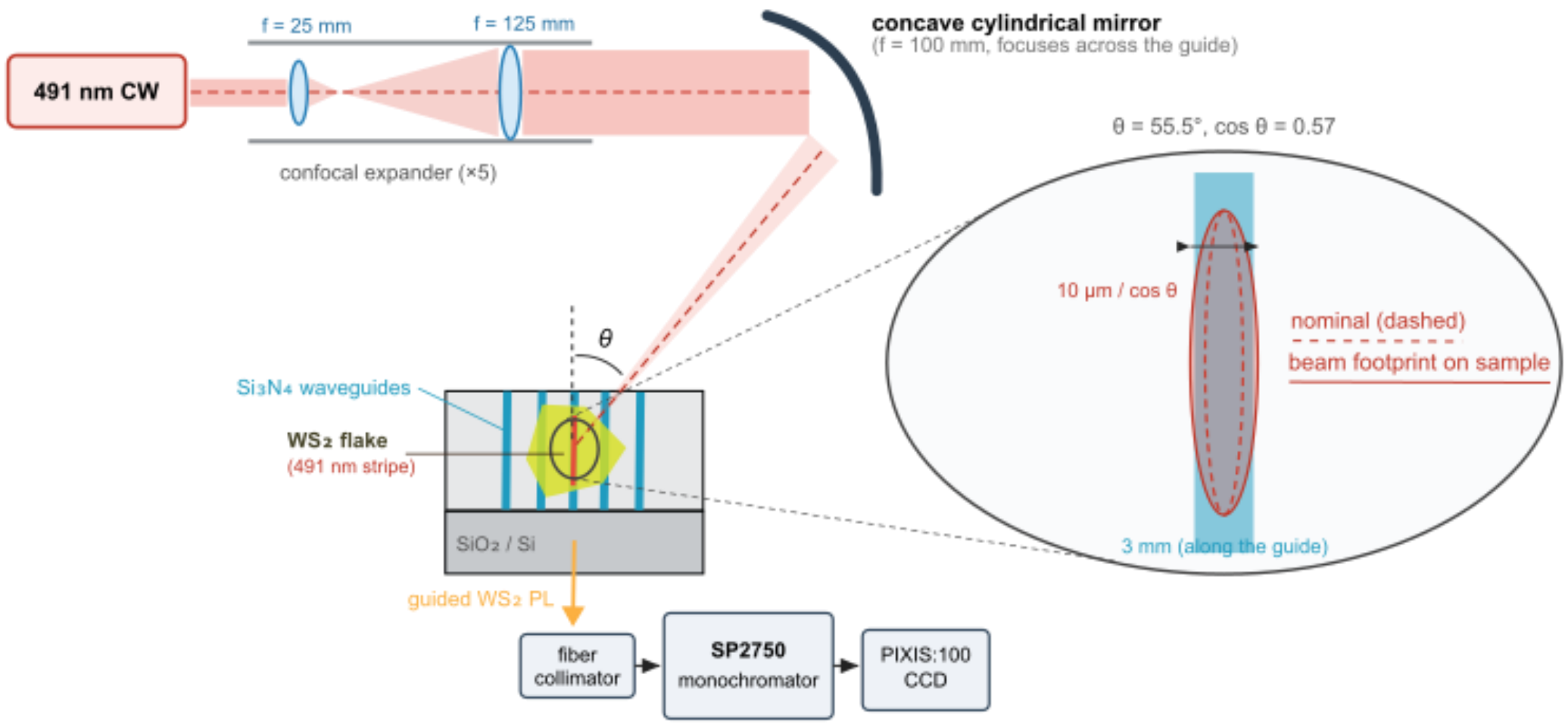

Figure S1. *Top-pumping excitation geometry. A 491 nm continuous-wave laser is expanded ×5 by a confocal two-lens telescope (f = 25 mm and f = 125 mm) and folded onto the chip by a concave cylindrical mirror (f = 100 mm), which focuses it along one axis into a 10 µm × 3 mm stripe oriented along the waveguide. The beam reaches the sample at 55.5° from the surface normal, so its elliptical footprint (zoom) has the 10 µm width stretched to 10 µm/cos ϑ and the on-sample photon-flux density reduced by cos ϑ = 0.57. Guided WS_2 PL from the output facet is collected by a fiber collimator and dispersed by an SP2750 monochromator onto a PIXIS:100 CCD.*

Conventional micro-photoluminescence (µ-PL) measurements were also performed using a top-excitation/top-collection geometry. In this configuration, a 532 nm continuous-wave laser was focused through a 100× microscope objective to a spot approximately 4 µm in diameter. A nominal laser power setting of 100% corresponds to 6 mW at the laser output and was adjusted using neutral-density filters. The same microscope objective was used to collect the µ-PL signal emitted by the TMD material, which was analyzed with a monochromator and a CCD.

All measurements were performed under ambient conditions.

S2. Power-dependent photoluminescence, exciton and trion contributions to the guided emission of WS_2

To better understand the guided WS_2 PL spectrum shown in Fig. 3d (orange curve), we compared two power-dependent PL series acquired on the same flake using the setups described in Section S1: (i) a conventional µ-PL series excited at 532 nm with powers ranging from 0.6 µW to 1.5 mW at the sample, and (ii) a guided top-pumping series excited with a 491 nm stripe beam with powers ranging from 12.5 mW to 125 mW at the sample.

In the µ-PL experiment, performed under normal-incidence excitation and therefore free from guided reabsorption effects, the neutral-exciton peak (X, 2.01 eV) dominates the emission spectrum at low excitation powers, whereas the negatively charged trion peak (X^-, 1.98 eV) becomes progressively more pronounced as the pump power increases (Fig. S2). Both contributions exhibit a sub-linear dependence on the incident photon flux Φ [photons s^{-1} cm^{-2}], with effective power-law exponents of $\alpha_x = 0.68$ and $\alpha_{x-} = 0.75$, respectively (Fig. S3a).[3] Because the trion emission increases slightly faster than the neutral-exciton emission, the relative trion contribution grows with increasing excitation density and eventually becomes comparable to, or larger than, the exciton contribution at the highest accessible powers. This trend suggests that the trion dominance would be further enhanced at even higher excitation densities.

The integrated guided PL signal also follows a sub-linear dependence on photon flux, with a power-law exponent $\alpha_{guided} = 0.69$ (Fig. S3b), comparable to those obtained from the µ-PL measurements. Although the nominal photon-flux densities shown in Figs. S3a and S3b are of similar magnitude, the guided-PL spectrum presented in Fig. 3d exhibits a substantially stronger trion contribution. A likely explanation is the higher absorption efficiency of WS_2 at the 491 nm excitation wavelength (2.53 eV) compared with 532 nm (2.33 eV). The larger absorption cross section at higher photon energies results in a higher density of photogenerated carriers for a given incident photon flux, thereby favoring trion formation and reinforcing the trion-dominated emission observed in the guided-PL spectrum.

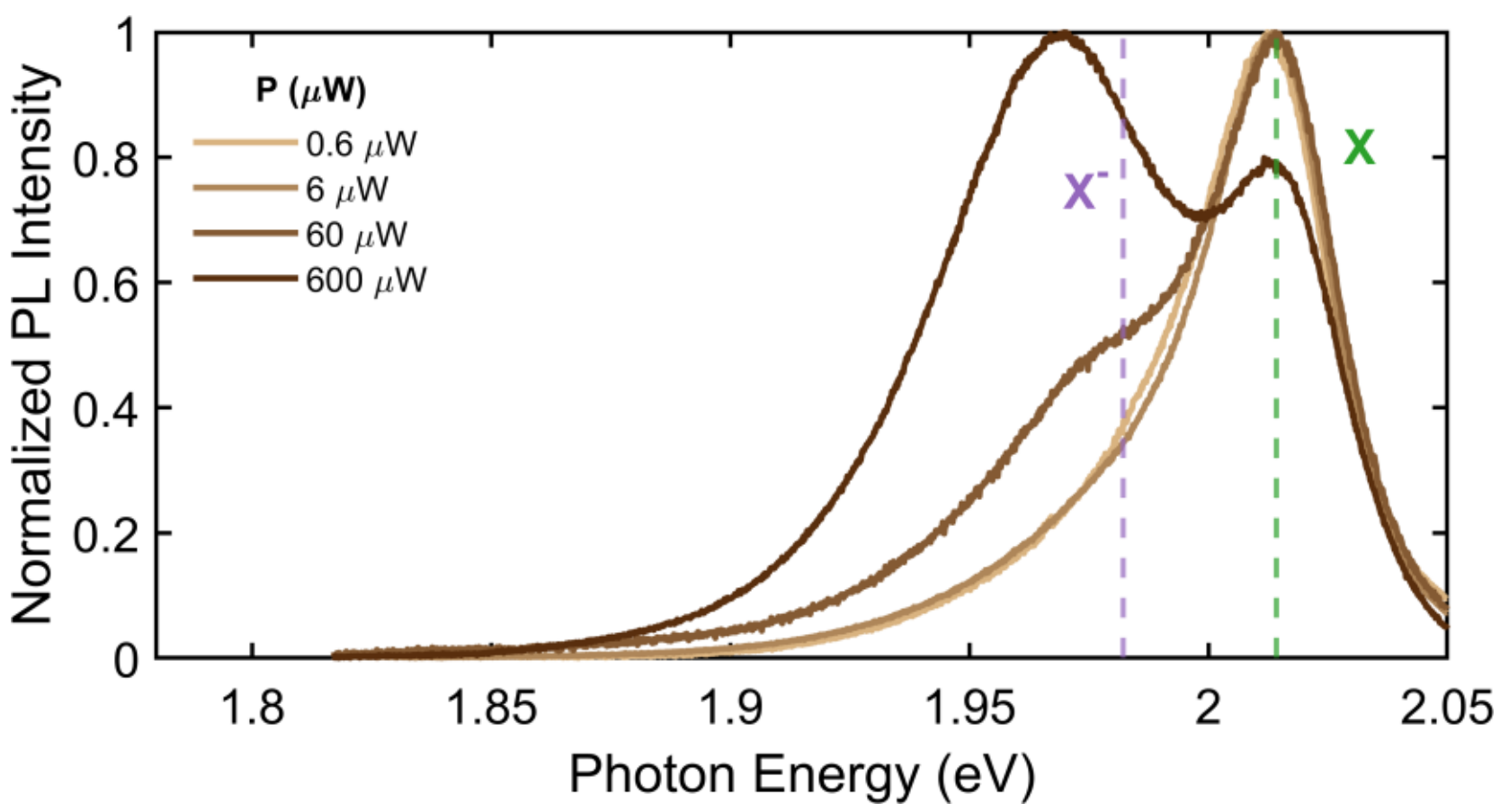

Figure S2. *Normalized micro-photoluminescence (µPL) spectra of WS_2 (532 nm, ×100 objective) for excitation powers 0.6 µW–1.5 mW. The neutral exciton (X, 2.01 eV) and charged trion (X^-, 1.98 eV) are resolved; the trion grows faster with power and dominates at high flux.*

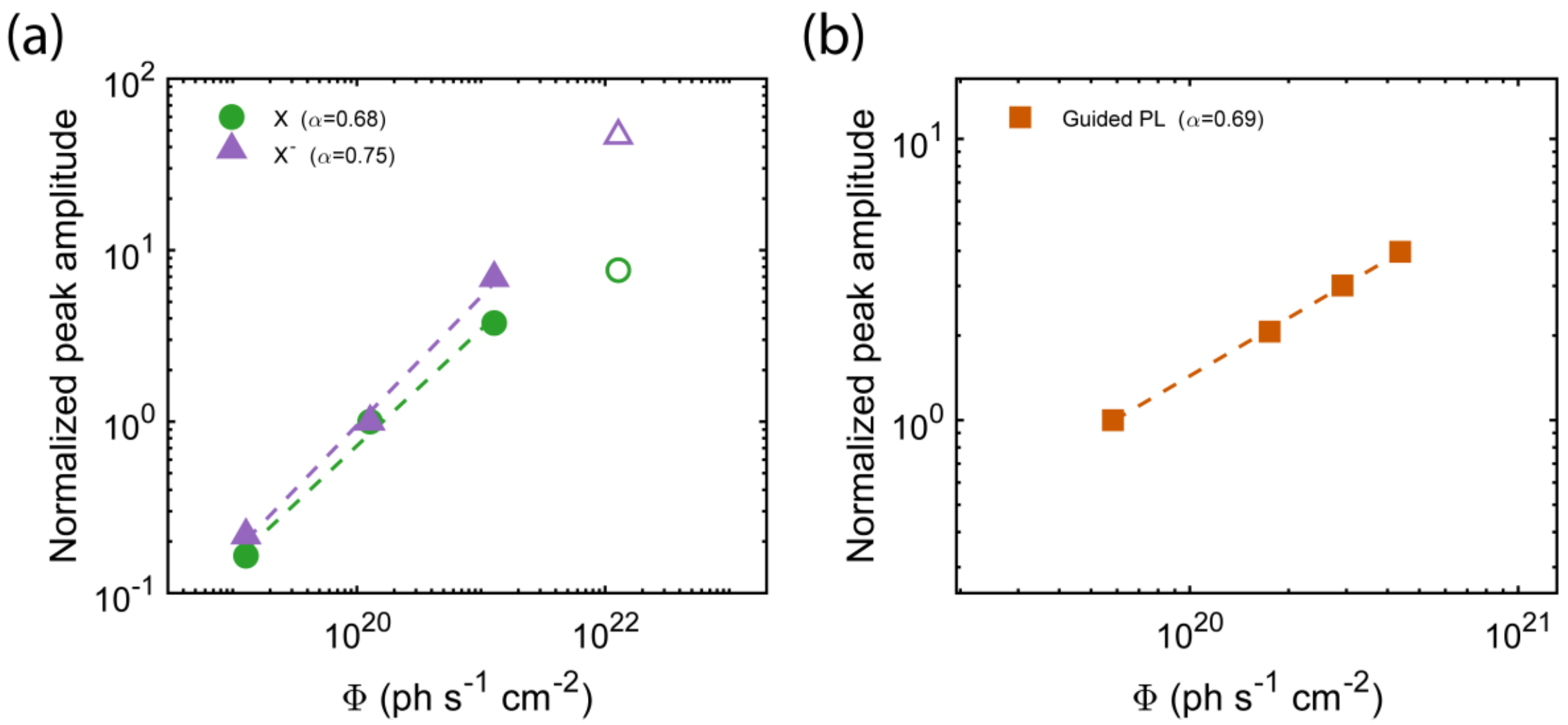

Figure S3. *Normalized PL amplitude versus photon-flux density Φ (log–log). (a) The μ-PL exciton (α_X = 0.68) and trion (α_{X^-} = 0.75) follow distinct sub-linear power laws; (b) the guided PL (α_{guided} = 0.69).*

S3. TMD coverage-length measurement

The attenuation spectra in the main text (Fig. 3b,c) are expressed per unit length, in dB cm^{-1}, obtained by normalizing the total absorbance accumulated over the TMD-covered section by the coverage length L. To measure L, a 405 nm beam was coupled from one facet and a 633 nm beam from the other, both strongly absorbed in the TMD-covered region. Each guided beam is therefore extinguished at the corresponding flake edge, and the dark gap between the two extinction points marks the covered length L (Fig. S4). Calibrating the image against the known 634 µm chip-block pitch converts this gap into an absolute length, giving L = 680 µm for the MoS_2 flake and L = 1.2 mm for the WS_2 flake.

The total absorbance A (in dB) yields an attenuation per unit length $\alpha\left(\frac{dB}{cm}\right)$.

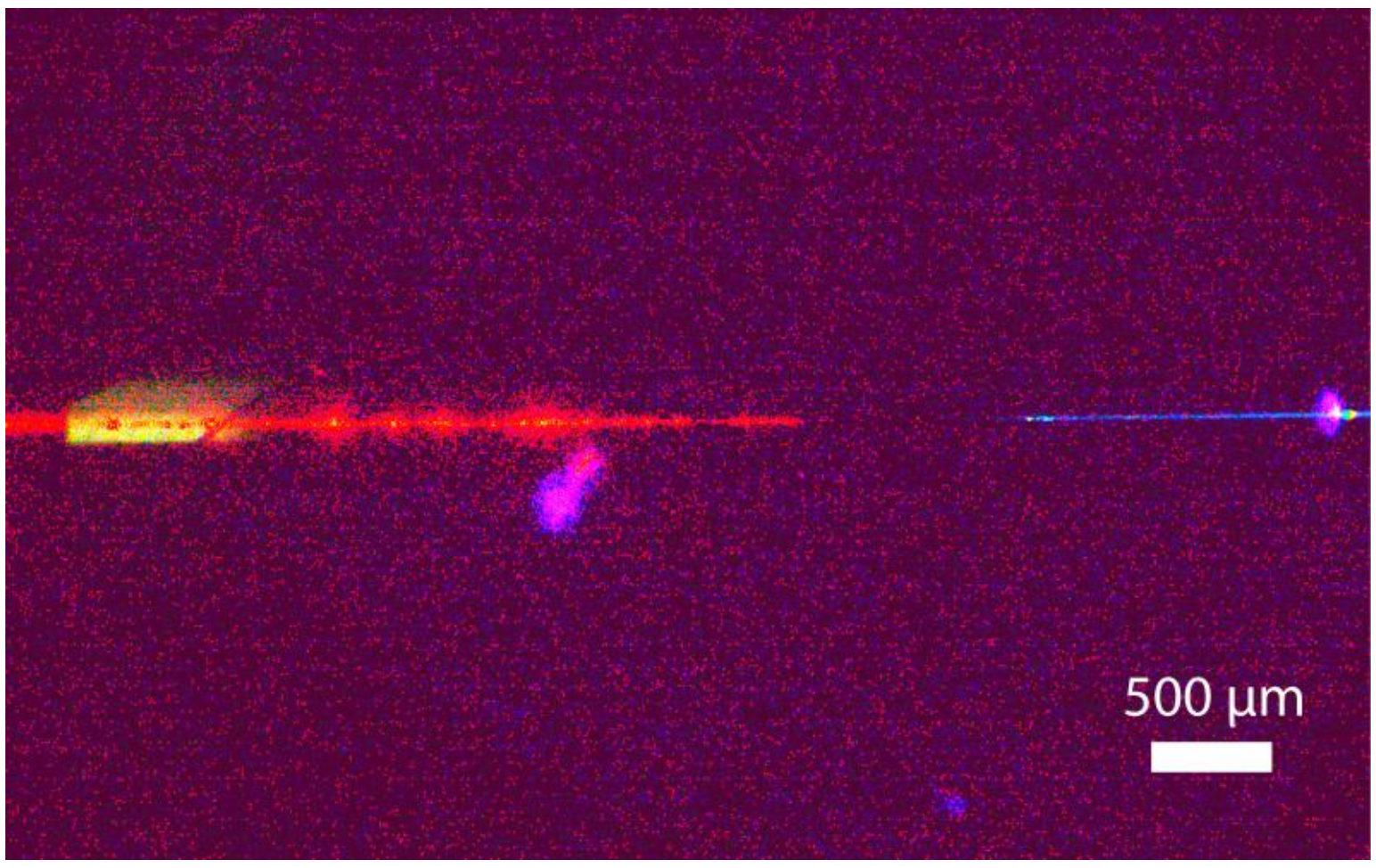

Figure S4. *Coverage-length measurement of the uniform MoS_2 flake on a 10-µm-wide waveguide, with light coupled simultaneously from both facets (405 nm from one side, 633 nm from the other), recorded at high exposure. The two counter-propagating guided beams and the dark gap between their extinction points are clearly visible; this gap marks the TMD-covered length. Using the 634 µm chip block pitch as the in-frame scale gives L = 680 µm for the MoS_2 flake; the WS_2 flake was measured analogously, giving L = 1.2 mm.*

S4. Waveguide losses

The scattered-light imaging technique is a non-destructive, top-view method commonly used to estimate propagation losses in optical waveguides and photonic integrated circuits. The technique relies on collecting the weak optical signal scattered out of the guiding plane as light propagates along the waveguide. Scattering originates from imperfections such as sidewall roughness, structural defects, and material inhomogeneities.

A high-resolution camera records the spatial distribution of the scattered light from above the chip (top panel of Fig. S5). The intensity profile along the propagation direction, z, is then extracted from the image (main panel of Fig. S5). Assuming that the scattered intensity is proportional to the guided optical power and that the scattering coefficient remains approximately constant along the waveguide, the propagation loss can be determined by fitting the measured intensity profile to an exponential decay:

$$I(z) = I_o e^{-\alpha z}$$

where α is the attenuation coefficient. The loss in decibels per centimeter (dB/cm) is then calculated as: $\alpha\left(\frac{dB}{cm}\right) = 4.34\alpha(cm^{-1})$. For the reference 10-µm-wide Si_3N_4 waveguide investigated here, the extracted propagation loss is about 6 dB/cm at 633 nm, corresponding to the spectral region where the TMD materials studied exhibit both absorption and photoluminescence. This value is more than one order of magnitude smaller than the attenuation measured in TMD-covered waveguides near the excitonic absorption edge, confirming that the optical losses reported in the main text are dominated by the interaction between the guided mode and the TMD layer rather than by intrinsic propagation losses of the Si_3N_4 platform.

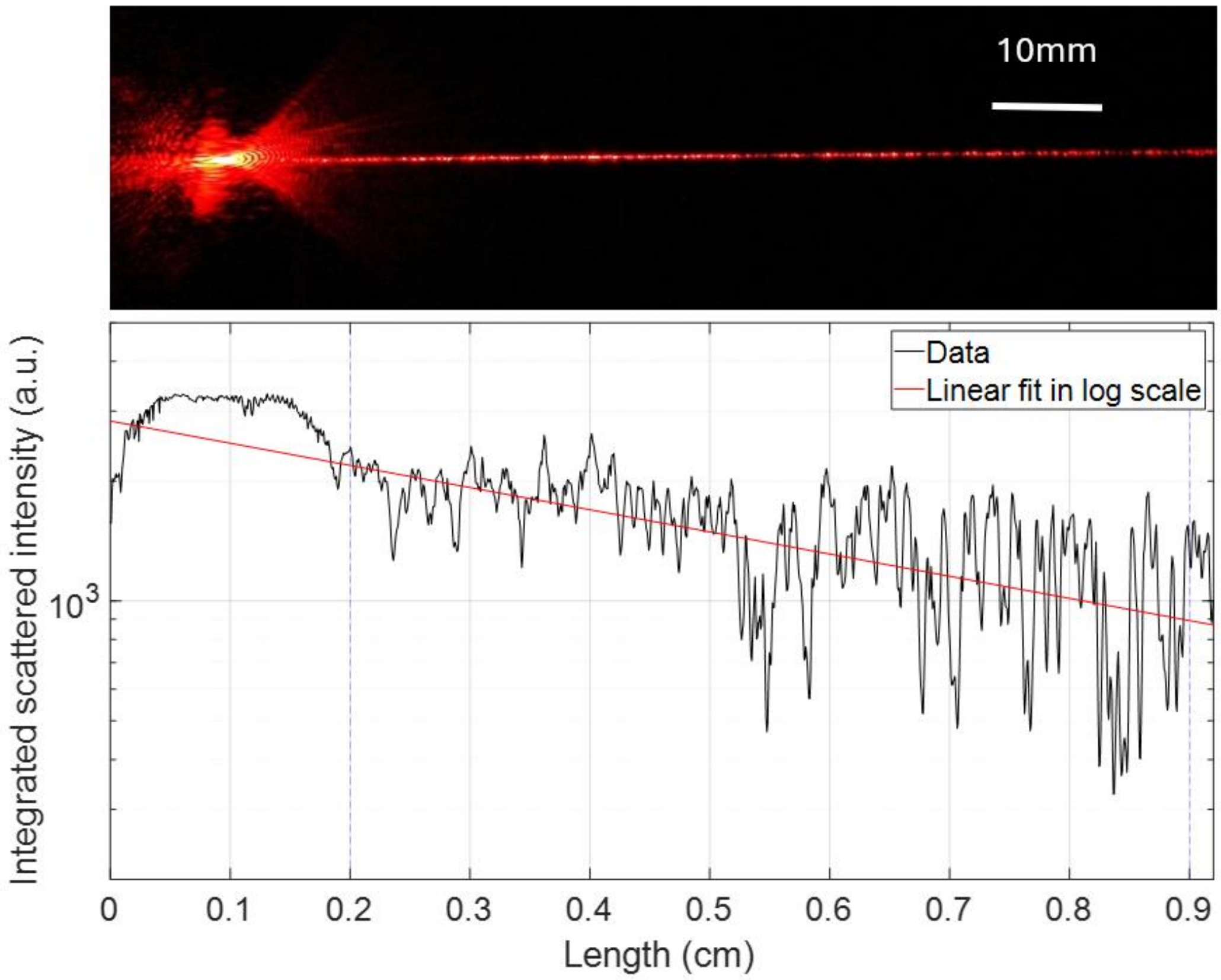

Figure S5. *Propagation loss measurement of a 10-µm-wide reference Si_3N_4 waveguide without TMD material, obtained using the scattered-light imaging technique. The top panel shows the scattered-light image acquired from above the chip, while the bottom panel presents the extracted intensity profile and exponential fit used to estimate the propagation loss (about 6dB/cm at 633 nm).*

S5. Polarization dependence

To investigate the possible influence of polarization on the extracted attenuation spectra, the guided photoluminescence emitted from the waveguide facet was collected in free space using a microscope objective. A linear polarizer was introduced in the collection path immediately before the monochromator and CCD detector to analyse two orthogonal linear polarization components of the emitted signal. Attenuation spectra were extracted from measurements performed without polarization selection and with the polarizer set to each of the two orthogonal orientations. As shown in Fig. S6, the three spectra exhibit very similar attenuation values and spectral evolution over the entire investigated energy range. These results indicate that, under the experimental conditions employed here, the extracted attenuation spectra are largely insensitive to the detected polarization state of the guided photoluminescence.

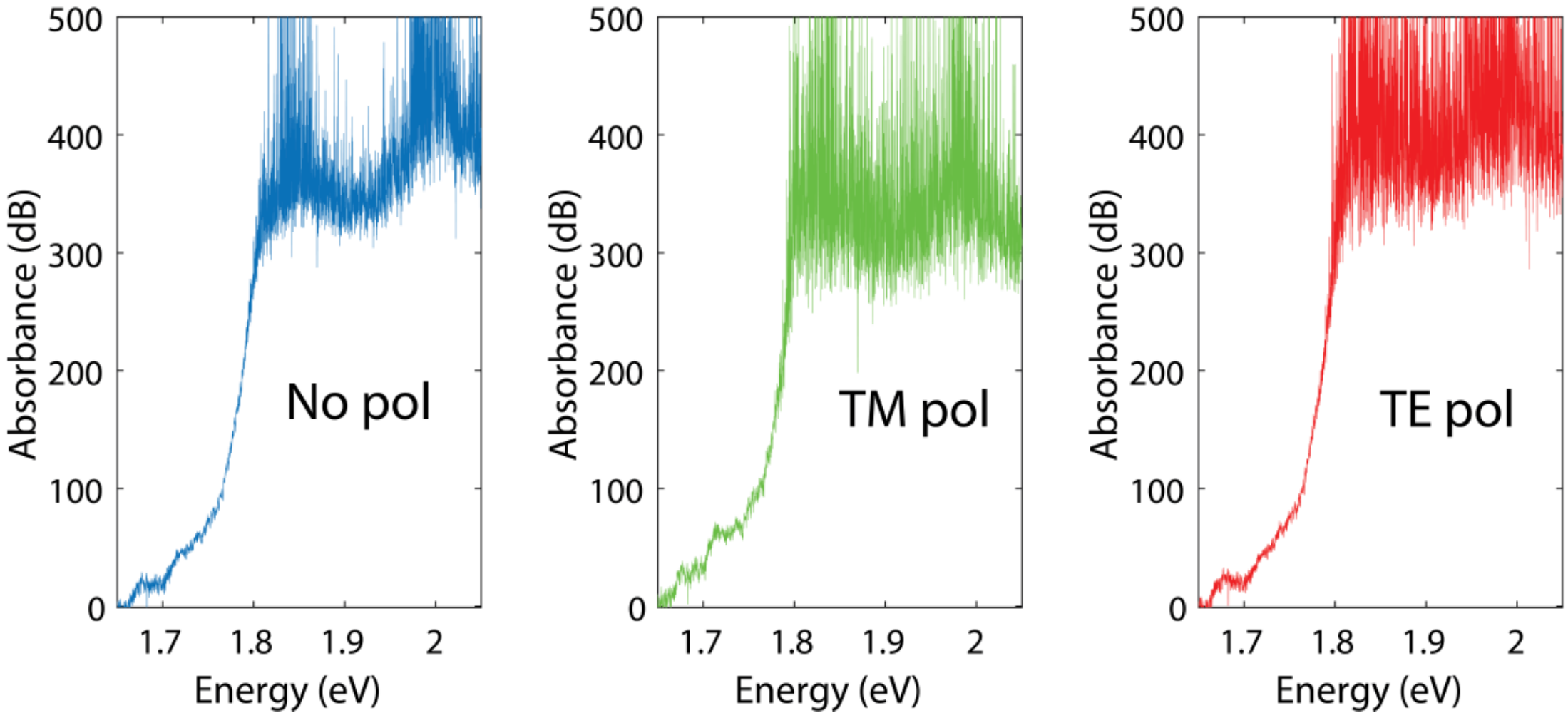

Figure S6. *Attenuation spectra extracted from guided photoluminescence measurements acquired without polarization selection (left panel) and with two orthogonal linear polarization settings in the detection path (middle and right panels). The guided photoluminescence emitted from the waveguide facet was collected in free space using a microscope objective, while a linear polarizer placed immediately before the monochromator and CCD detector was used for polarization analysis. The three attenuation spectra exhibit comparable values and similar spectral evolution, indicating that the extracted absorption losses are essentially independent of the detected polarization state under the experimental conditions employed in this work.*

References

(1) Khosrofian, J. M.; Garetz, B. A. Measurement of a Gaussian Laser Beam Diameter through the Direct Inversion of Knife-Edge Data. *Appl. Opt.* **1983**, *22* (21), 3406. https://doi.org/10.1364/ao.22.003406

(2) De Araújo, M. A. C.; Silva, R.; De Lima, E.; Pereira, D. P.; De Oliveira, P. C. Measurement of Gaussian Laser Beam Radius Using the Knife-Edge Technique: Improvement on Data Analysis. *Appl. Opt.* **2009**, *48* (2), 393–396. https://doi.org/10.1364/AO.48.000393

(3) Plechinger, G.; Nagler, P.; Arora, A.; Schmidt, R.; Chernikov, A.; Del Águila, A. G.; Christianen, P. C. M.; Bratschitsch, R.; Schüller, C.; Korn, T. Trion Fine Structure and Coupled Spin-Valley Dynamics in Monolayer Tungsten Disulfide. *Nat. Commun.* **2016**, *7*, 12715. https://doi.org/10.1038/ncomms12715
